# GLP-1R Agonists for Weight Loss in Psychiatric Disorders: A Systematic Review and Meta-analysis

**DOI:** 10.1210/jendso/bvaf150

**Published:** 2025-11-11

**Authors:** Klara Müller Alves, Manoela Teixeira da Silva, Caroline Kaercher Kramer, Luciana Verçoza Viana

**Affiliations:** Post-Graduate Program in Medical Sciences, Endocrinology, Federal University of Rio Grande do Sul, Porto Alegre 90035-903, Brazil; Post-Graduate Program in Medical Sciences, Endocrinology, Federal University of Rio Grande do Sul, Porto Alegre 90035-903, Brazil; Division of Endocrinology, University of Toronto, Toronto, ON M5T 3L9, Canada; Post-Graduate Program in Medical Sciences, Endocrinology, Federal University of Rio Grande do Sul, Porto Alegre 90035-903, Brazil; Division of Endocrinology, Hospital de Clínicas de Porto Alegre, Porto Alegre 90035-903, Brazil

**Keywords:** GLP-1 receptor agonists, liraglutide, semaglutide, weight loss, psychiatric disorders, obesity

## Abstract

**Context:**

Individuals with psychiatric disorders have a higher prevalence of obesity, partly because of the use of psychotropic medications. Safe and effective pharmacological interventions for weight management in this population are needed.

**Objective:**

To assess the efficacy and safety of glucagon-like peptide-1 receptor agonists (GLP-1Ras) in individuals with psychiatric disorders and obesity/overweight through a systematic review and meta-analysis, focusing on weight and metabolic outcomes.

**Data Sources:**

We searched PubMed, Embase, Cochrane CENTRAL, and ClinicalTrials.gov until May 2025 for observational studies that evaluated GLP-1RAs in this population.

**Study Selection:**

Studies including adults with psychiatric disorders and obesity treated with GLP-1RAs were eligible; 10 randomized controlled trials met the inclusion criteria.

**Data Extraction:**

Two reviewers independently extracted the data and assessed the risk of bias using the Cochrane RoB 2 tool. Outcomes included weight, body mass index (BMI), waist circumference, glucose level, systolic blood pressure, total cholesterol, high-density lipoprotein cholesterol, triglycerides, and adverse events.

**Data Synthesis:**

GLP-1RAs significantly reduced body weight [mean difference (MD) −5.03 kg; 95% confidence interval (CI): −6.04 to −4.01)], BMI (MD −1.59 kg/m²; 95% CI: −2 to −1.18), waist circumference (MD −3.4 cm 95% CI: −4.83 to−1.97), and fasting glucose (MD −0.29 mmol/L; 95% CI: −0.53 to −0.05) compared to controls. Gastrointestinal side effects were more frequent but generally mild and did not increase discontinuation rates.

**Conclusion:**

GLP-1RAs are effective and well-tolerated for managing obesity in psychiatric populations, offering significant weight and metabolic benefits. Further studies are needed to evaluate newer agents, such as semaglutide and tirzepatide, particularly in longer trials with standardized protocols.

Individuals with psychiatric disorders exhibit higher mortality rates than those in the general population [1, 2]. A considerable proportion of this increase in mortality is linked to obesity and associated health complications [3].

The prevalence of obesity is notably higher among individuals with psychiatric disorders than among those without psychiatric disorders [4, 5]. This complex endocrine-metabolic condition, particularly when associated with metabolic syndrome, is a well-established risk factor for the development of chronic diseases, such as type 2 diabetes and cardiovascular disease [2, 4, 6]. Multiple factors contribute to the elevated risk of metabolic disorders in individuals with psychiatric conditions. These include poor dietary habits (eg, monotonous, nutrient-poor eating patterns), reduced health self-management, low physical activity (often due to diminished motivation linked to the psychiatric condition itself), and treatment with specific psychiatric medications, such as antipsychotics and mood stabilizers, which are known to promote weight gain [3, 4, 7, 8].

Current evidence-based approaches to obesity management incorporate a combination of interventions, including reduced caloric intake, regular physical activity, cognitive behavioral therapy, and pharmacotherapy [9]. There is growing scientific interest in the potential role of glucagon-like peptide-1 receptor agonists (GLP-1RAs) as an effective therapeutic strategy for sustainable weight loss in patients with psychiatric disorders.

GLP-1RAs have been shown to promote greater weight loss than lifestyle intervention [9-11]. In patients without psychiatric disease, weight loss from drugs such as liraglutide, exenatide, dulaglutide, and semaglutide ranges between −5.3 kg and −12.3 kg [12-16] and around −24 kg for tirzepatide [17, 18]. Nevertheless, given the recency of these agents, the efficacy, safety, and overall applicability of this strategy for psychiatric patients remain uncertain, with limited accessible randomized controlled trials (RCTs), and the available evidence is insufficient to conclusively support the routine clinical implementation of GLP-1RAs or to fully define their safety profile within this vulnerable population [19].

In light of the emerging role of incretin-based therapies in addressing obesity in a specific population, this systematic review and meta-analysis aimed to evaluate the effects of GLP-1RAs on weight changes and clinical and metabolic outcomes in individuals with psychiatric disorders and obesity/overweight.

## Methods

The protocol for this review is available on PROSPERO (CRD42023418437).

We searched the literature in the US National Library of Medicine (PubMed), Latin American and Caribbean Literature in Health Sciences, Embase, Cochrane Library, Web of Science, Scopus, and the registry of multiple trials, including ClinicalTrials.gov, from the National Institutes of Health. We used a combination of text words (tw) and MeSH headings (mh), including Glucagon-Like Peptide 1[mh] OR GLP-1[tw] AND Mental Disorders[mh] OR Mental Disorder*[tw] OR Behavior Disorder*[tw] OR Mental Illness[tw] OR Psychiatric Diagnosis*[tw] OR Psychiatric Disease*[tw] OR Psychiatric Disorder* AND Overweight[mh] OR Overweight[tw] OR Weight Gain. The search strategies for this review were developed with the assistance of an experienced librarian. Additionally, we manually searched the references of the included articles published in English.

### Trial Selection

The PRISMA guidelines were followed [20]. Two investigators (K.M.A. and M.T.S.) examined the titles and abstracts and independently examined the full-text reports and abstracts. The inclusion criteria were as follows: (1) adults aged ≥18 years and (2) diagnosis of any psychiatric disorder as stated in the papers, (3) people with obesity or overweight [body mass index (BMI) > 25.0 kg/m²]; (4) use of any GLP-1 agonist as an intervention, (5) randomized controlled trials or observational studies, (6) participants were allocated to a GLP-1RA intervention or control group (placebo or other medications). The exclusion criteria were as follows: (1) case series, case studies, and conference/congress abstracts with insufficient data and (2) full content access was not possible. Only studies published between January 1, 2000, and May 6, 2025, were selected for review. This time frame was selected because of the emergence of GLP-1RAs in clinical research in the early 2000s. Any disagreements were resolved by consensus with L.V.V.

### Outcomes

The outcomes were (1) changes in body weight and (2) BMI. Secondary outcomes were (1) cardiometabolic risk factors such as fasting glucose, total cholesterol, high-density lipoprotein, systolic blood pressure, triglycerides, and waist circumference and (2) adverse effects.

### Data Extraction

Data were extracted independently by K.M.A. and M.T.S. using a structured and pretested form. L.V.V. provided assistance as an adjudicator in cases of disagreements. Data extraction included changes in the outcomes; incidence of adverse events and information regarding sample description, such as age, sex, race, comorbidities, psychiatric diagnosis, medications in use, and sample size; and study characteristics, such as study design, duration of intervention, type of GLP-1RA used as intervention, percentage of loss to follow-up, the year it occurred, and where it took place.

### Risk of Bias Assessment

Two researchers (K.M.A. and M.T.S.) independently assessed the methodological quality of the included studies. The risk of bias in intervention studies was assessed using methods developed by the Cochrane Group, the RoB 2.0 (Revised Cochrane Risk-of-Bias) tool [21]. The following bias domains were evaluated as high risk, low risk, or having some concerns: (1) random sequence generation (selection bias), (2) allocation concealment (selection bias), (3) blinding of participants and personnel (performance bias), (4) blinding of outcome assessment (detection bias), (5) incomplete outcome data (attrition bias), (6) selective reporting (reporting bias), and (7) other possible sources of bias. For a trial to be classified as “low risk,” all domains needed to be assessed as low risk, with the exception of participant blinding. The Newcastle–Ottawa Quality Assessment Scale was used for observational studies. A funnel plot was used to assess publication bias in the case of a sufficient number of studies (n ≥ 10).

### Statistical Analyses

We examined the effect of GLP-1RAs on changes in weight and all other outcomes compared with those in the control arm. Our meta-analysis used a random-effects model to calculate the standardized mean differences (MD) with SD for continuous outcomes and relative risk for categorical variables. Certain unit adjustments and central tendency measure conversions were necessary to ensure data comparability according to Cochrane's Handbook [22]. Results were considered significant when *P* < .05 and were reported with 95% confidence intervals (CIs). In addition, the chi-square test (Cochrane Q test) was used to assess the statistical heterogeneity of the included studies. I^2^ represents the percentage of variation across studies due to heterogeneity rather than chance. Significant heterogeneity was reflected by I^2^ > 50% and a *P*-value < .1. Data details are available as supplemental material [23].

A network meta-analysis was conducted to draw direct and indirect comparisons between the treatment groups. The analysis was performed using a frequentist graph-based approach, which enabled both direct and indirect comparisons between the treatments. Common-effect and random-effects models were adjusted using the Der Simonian-Laird estimator to estimate the between-study variance. All analyses were performed using R (version 4.3.3).

## Results

### Results of Study Selection and Characteristics

**Figure 1.**
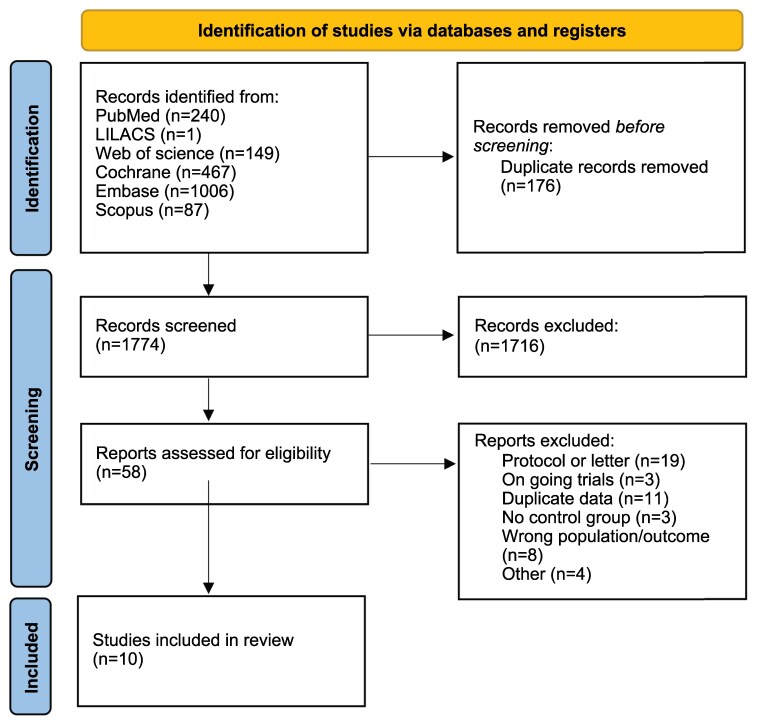
Flow diagram of literature search to identify randomized controlled trials of glucagon-like peptide-1 receptor agonists for weight loss in psychiatric patients.

Of the 1950 records identified, 1716 were excluded based on title and abstract, leaving 58 studies for full-text evaluation; ultimately, 10 RCTs were included in this systematic review (Fig. 1).

Table 1 shows the characteristics of the 10 studies included in this systematic review.

**Table 1.** Studies summary of GLP-1RA in individuals with psychiatric disorders and obesity

Author, year	Country	Number of randomized subjects	Study design	Pathology	GLP-1RA	Psychiatric medication	Intervention/expousure	Control/Comparator	Study duration
Robert et al, 2015 [24]	Malaysia	42	RCT	Binge eating	Liraglutide	Not reported	Liraglutide 1.8 mg/d + diet + exercise	Diet + exercise	12 weeks
Ishøy et al, 2017 [25]	Denmark	40	RCT	Schizophrenia	Exenatide	Antipsychotics	Exenatide 2 mg/week	Placebo	16 weeks
Larsen et al, 2017 [26]	Denmark	103	RCT	Schizophrenia	Liraglutide	Clozapine, olanzapine	Liraglutide 1.8 mg/day	Placebo	16 weeks
Siskind et al, 2018 [27]	Australia	28	RCT	Schizophrenia	Exenatide	Clozapine	Exenatide 2 mg/week + usual care	Placebo	24 weeks
Da Porto et al, 2020 [28]	Italy	60	RCT	Binge eating	Dulaglutide	Not reported	Dulaglutide 150 mg/week	Gliclazida 60 mg + metformin 2-3 g/day	12 weeks
Whicher et al, 2021 [29]	United Kingdom	47	RCT	Schizophrenia and psychosis	Liraglutide	Antipsychotics	Liraglutide 3 mg/day + orientations	Standardized written information about healthy eating, physical activity, and smoking	24 weeks
Allison et al, 2022 [30]	United States	37	RCT	Binge eating	Liraglutide	Not reported	Liraglutide 3 mg/day	Placebo	17 weeks
McElroy et al, 2024 [31]	United States	60	RCT	Bipolar disorder	Liraglutide	Mood stabilizers, antipsychotics, antidepressants and anxiolytics	Liraglutide 3 mg/day + nutrition and lifestyle modification	Nutrition and lifestyle modification counseling following the 2015-2020 Dietary Guidelines for Americans	24 weeks
Badulescu et al, 2025 [32]	Canada	72	RCT	Major depressive disorder	Oral semaglutide	Benzodiazepines, antipsychotics, antidepressants, psychostimulants, bupropion.	Oral semaglutide 14 mg/day	Placebo	16 weeks
Patino et al, 2025 [33]	United States	54	RCT	Schizophrenia, bipolar disorder, and severe depression	Exenatide	Olanzapine	Exenatide 10 mcg/2xday	Placebo	16 weeks

Abbreviations: GLP-1RA, glucagon-like peptide-1 receptor agonist; RCT, randomized controlled trial.

Five randomized controlled trials compared liraglutide with usual care [24, 26, 29-31], 3 RCTs compared exenatide with usual care [25, 27, 33], 1 compared oral semaglutide [32] with usual care, and the remaining trial compared dulaglutide with gliclazide 60 mg + metformin 2-3 g/day [28]. The dulaglutide trial, which involved participants with obesity and diabetes, was excluded from the meta-analysis because the control group received active medication rather than a placebo.

The psychiatric disorders diagnosed in the studies were schizophrenia or schizoaffective disorder (n = 4), bipolar disorder (n = 2), psychosis (n = 1), major depressive disorder (n = 2), and binge-eating disorder (n = 3). Some trials included participants with multiple psychiatric conditions (n = 2). Type 2 diabetes was the most common nonpsychiatric comorbidity (∼15%), although 4 RCTs excluded individuals with diabetes mellitus. The most frequently used psychiatric medications among the participants included mood stabilizers (eg, carbamazepine, lamotrigine, lithium, and valproate), antipsychotics (first and second generation), antidepressants, and anxiolytics (eg, benzodiazepines, gabapentin, pregabalin, and buspirone).

The trials were conducted in America (n = 4), Europe (n = 4), Asia (n = 1), and Australia (n = 1), providing data from 543 individuals. Approximately 52.8% of participants were women. The mean age ranged from 34.4 to 55.1 years. Baseline body weight averaged 103.8 ± 18.4 kg in the intervention group and 103.2 ± 19.0 kg in the control group. Baseline BMI was 36.0 ± 6.1 kg/m² in the intervention group and 36.3 ± 5.1 kg/m² in the control group. The mean study duration was 17.7 weeks (12-24 weeks).

### Outcomes

#### Weight loss

**Figure 2.**
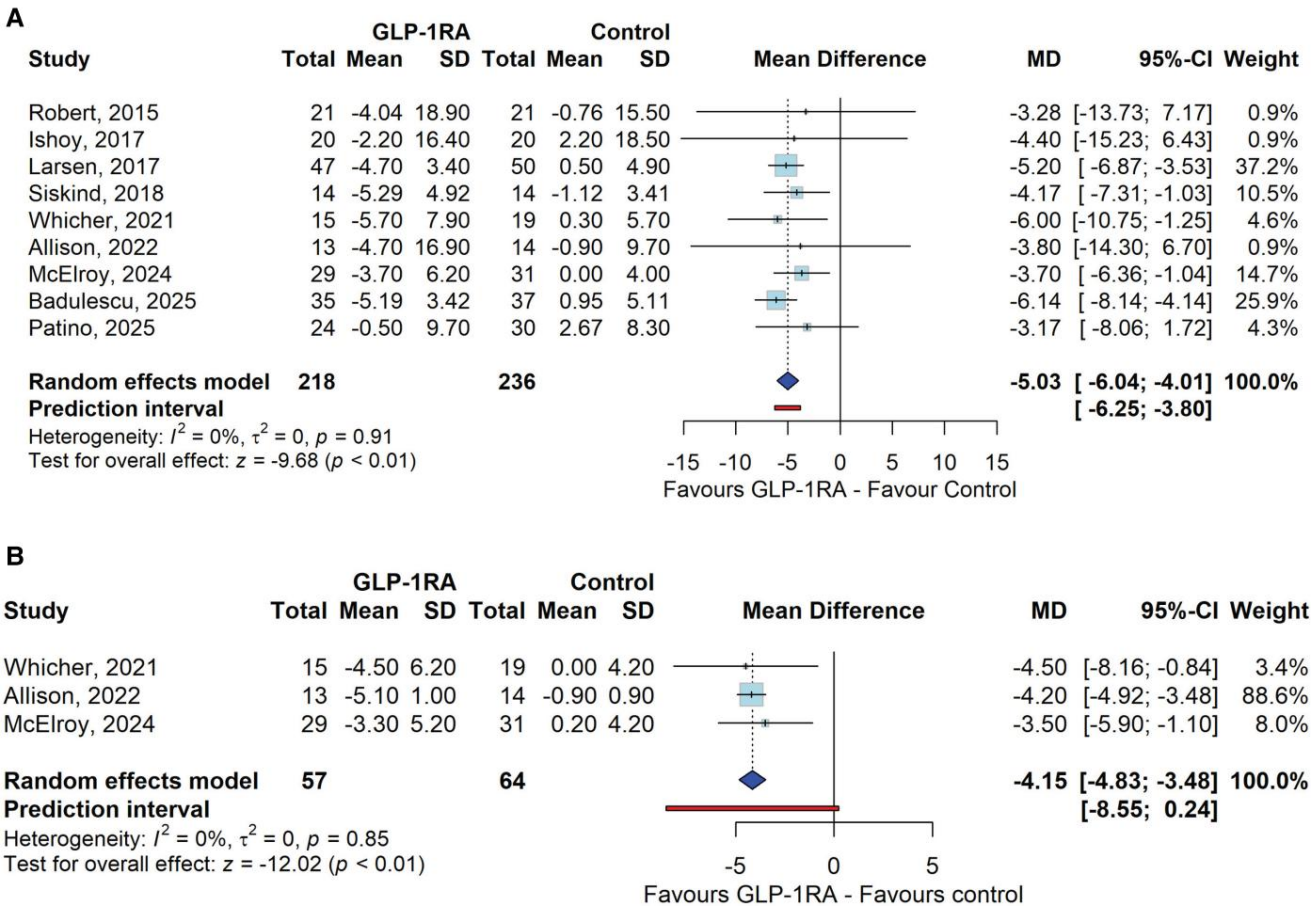
Effect of GLP-1RAs compared to control on body weight (A) and percent of weight loss (B) among individuals with psychiatric disorders and obesity. The findings of each study are represented by a single square. The diamond represents the overall effect estimate of the meta-analysis. Abbreviations: CI, confidence interval; GLP-1RA, glucagon-like peptide-1 receptor Agonist; MD, mean difference.

Pooling of the data from the 9 RCTs showed that the use of GLP-1RAs compared to control induced weight reduction of 5.03 kg (95% CI: −6.04, −4.01, I² = 0%), as shown in Fig. 2. Similarly, the percentage of weight loss was greater in the GLP-1RA group (MD = −4.15%, 95% CI: −4.83 to −3.48; I² = 0%) (Fig. 2).

BMI reduction was also significant in the GLP-1RA group, with a mean decrease of −1.59 kg/m² (95% CI: −2.00 to −1.18; I² = 0%) (Fig. S2.3) [23].

Subanalysis by type of administered GLP-1RA showed that liraglutide promoted a weight loss of 4.83 kg (95% CI: −6.17 to −3.50, I² = 0%) and a BMI improvement of −1.30 kg/m² (95% CI: −1.92 to −0.69, I² = 0%). Exenatide promoted 3.91 kg of weight loss (95% CI: −6.47 to −1.34, I² = 0%) and a −1.25 kg/m² improvement in BMI (95% CI: −2.12 to −0.37, I² = 0%). Semaglutide promoted a weight loss of 6.14 kg (95% CI: −8.14 to −4.14) and a BMI improvement of −2.16 kg/m² (95% CI: −2.85 to −1.47) (Fig. S3.1 A and B) [23].

In a subgroup analysis of patients with schizophrenia, GLP-1RA led to weight loss −5.05 kg (95% CI: −6.45 to −3.66, I² = 0%) and BMI −1.43 kg/m² (95% CI: −2.25 to −0.62, I² = 0%) (Fig. S3.2 A and B) [23].

A network meta-analysis confirmed that semaglutide showed the greatest mean reduction in body weight compared with placebo, with a mean difference of −6.14 kg (95% CI: −8.18 to −4.10; *P* < .001). Liraglutide was also significantly superior to placebo, with a mean difference of −5.01 kg (95% CI: −6.32 to −3.70; *P* < .001). Although exenatide demonstrated a reduction in weight compared with placebo (MD = −4.03 kg; 95% CI: −8.65 to 0.60), the result was not statistically significant (*P* = .088). Pairwise comparisons between the active treatments revealed no significant difference. The global inconsistency test yielded a *P*-value of .99, indicating no inconsistency and high reliability of the results (League Table) [23].

#### Secondary outcomes

**Figure 3.**
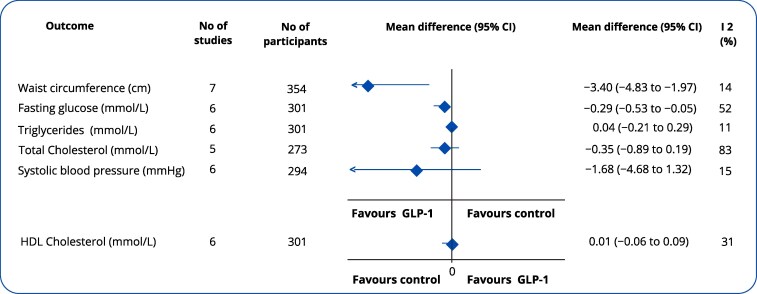
Effect of glucagon-like peptide-1 receptor agonists compared to control on waist circumference, fasting glucose, triglycerides, total cholesterol, systolic blood pressure, and high-density lipoprotein cholesterol among individuals with psychiatric disorders and obesity. The diamond represents the overall effect estimate of the meta-analysis. Abbreviation: CI, confidence interval.

GLP-1RA treatment, compared to usual care, led to a significant reduction in waist circumference (MD = −3.40 cm, 95% CI: −4.83 to −1.97, I² = 14%) and fasting glucose (MD = −0.29 mmol/L, 95% CI: −0.53 to −0.05, I² = 52%) (Fig. 3). A subanalysis by type of administered GLP-1RA showed that liraglutide led to a fasting glucose decrease of 0.33 mmol/L (95% CI: −0.54 to −0.12, I² = 26%) reduction, while exenatide resulted in a 0.26 mmol/L (95% CI: −1.09 to 0.56, I² = 80%) reduction (Fig. S3.1 D) [23].

Treatment with GLP-1RA compared to the control did not result in a significant decrease in total cholesterol (MD = −0.35, 95% CI: −0.89 to 0.19, I² = 83%; *P* = .20), systolic blood pressure (MD = −1.68, 95% CI: −4.68, 1.32, I² = 15%; *P* = .27), and triglycerides (MD = 0.04, 95% CI: −0.21 to 0.29, I² = 11%; *P* = .74) or an increase in high-density lipoprotein cholesterol (MD = 0.01, 95% CI: −0.06 to 0.09, I² = 31%; *P* = .71) (Fig. 3).

#### Adverse events

Data from 374 individuals were available for adverse events. The evaluation of these events in the trials was primarily based on individual reports. Gastrointestinal tract side effects were the most frequent. Meta-analyses indicated that GLP-1RA treatment was associated with a relative risk of 1.84 for diarrhea (95% CI: 1.11-3.07), 2.01 for nausea (95% CI: 1.41-2.88), 2.46 for vomiting (95% CI: 1.28-4.72), and 2.69 for constipation (95% CI: 1.34-5.42) (Table 2).

**Table 2.** Meta-analysis of incidence (%) and RR of adverse events in RCTs

Adverse effect	Intervention	No. studies	n	Incidence %	95% CI	I² (%)
Diarrhea	GLP-1RA	7	184	24.32	0.16-0.39	65.4
	Placebo	6	175	10.85	0.07-0.16	0
Nausea	GLP-1RA	8	205	50.24	0.31-0.61	70.6
	Placebo	7	196	23.46	0.11-0.32	40.9
Vomiting	GLP-1RA	6	155	29.67	0.20-0.39	34.1
	Placebo	5	144	10.41	0.04-0.18	0
Constipation	GLP-1RA	4	131	32.06	0.24-0.40	15.3
	Placebo	4	138	10.40	0.04-0.21	49.9

Adverse effect	Intervention	No. studies	n	RR	95% CI	I² (%)
Diarrhea		6	345	1.84	1.11-3.07	0.2
Nausea		7	387	2.01	1.41-2.88	15.5
Vomiting		5	285	2.46	1.28-4.72	4.2
Constipation		4	269	2.69	1.34-5.42	19.7

Abbreviations: CI, confidence interval; RR, relative risk.

#### Risk of bias assessment

**Figure 4.**
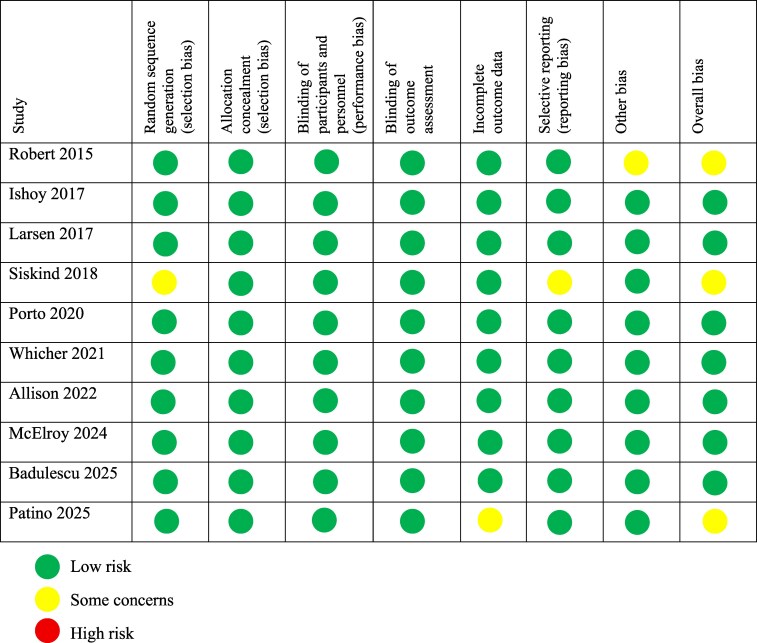
Risk of bias assessment of all randomized controlled trials.

The revised Cochrane tool for assessing the risk of bias in RCTs [20] identified a low risk of bias in 7 studies [24, 26, 27, 29, 30, 32, 33], while 3 [23, 25, 31] presented some concerns of bias, particularly due to incomplete outcome data and selective reporting. Overall, the included RCTs presented a low risk of bias, supporting the reliability of the findings (Fig. 4).

## Discussion

In the present study, GLP-1RAs were associated with a mean weight loss of 5.03 kg, corresponding to a 4.15% reduction in body weight; additional metabolic benefits included reductions in BMI (−1.59 kg/m²), waist circumference (−3.40 cm), and fasting glucose levels (−0.29 mmol/L), compared to individuals not receiving GLP-1RAs among patients with mood disorders, schizophrenia, and binge-eating disorder. The use of GLP-1RAs is associated with a higher incidence of gastrointestinal side effects, although no serious adverse events have been reported.

Recent systematic reviews [34-39] have evaluated the use of GLP-1RAs in various psychiatric conditions. Menon et al reported positive outcomes in addressing psychotropic-induced weight gain, suggesting that GLP-1RAs may directly modulate the metabolic pathways and neural circuits involved in hunger and satiety. Similarly, Vasiliu's review, which encompassed both clinical and preclinical studies, further supports the metabolic benefits of these agents. Our findings align with those of Khaity et al, who examined GLP-1RA effects in patients with schizophrenia, and Radkhah et al, who reported similar benefits in individuals with eating disorders.

GLP-1RAs appear to exert metabolic effects through multiple mechanisms. They slow gastric emptying and promote satiety through gastrointestinal actions. In addition, there is growing evidence that GLP-1RAs modulate the mesolimbic dopamine system, a neural circuit involved in reward processing, leading to decreased responsiveness to highly palatable, ultraprocessed foods and reduced food-related impulsivity and addiction-like behaviors [40, 41]. These mechanisms may also underlie the reported reductions in alcohol consumption among individuals with alcohol use disorder [42-44]. Supporting this broader neuropsychiatric impact, the findings of Pierret et al’s review and meta-analysis indicate that treatment with GLP-1 receptor agonists in obesity/overweight is linked to enhanced mental well-being and quality of life, along with established benefits for physical health [45].

Prior to the advent of GLP-1RAs, other pharmacological agents such as topiramate [46-48], topiramate/phentermine [49], and naltrexone/bupropion [50-52] had been investigated for weight management in psychiatric populations. While these agents demonstrate modest weight reduction, their adverse effect profiles limit their widespread clinical use. Adjunctive metformin has been the most extensively studied pharmacotherapy and is recommended as a level 1 treatment for mitigating weight gain in patients with psychiatric disorders receiving antipsychotic therapy, as it has demonstrated efficacy in preventing and reducing weight gain, BMI increases, and insulin resistance in both antipsychotic-in-use individuals and those with established weight and metabolic disturbances [53-55]. Metformin may exert these effects through its action on the incretin axis, upregulating GLP-1 receptor expression and increasing circulating GLP-1 levels, which further supports GLP-1RAs role as a promising adjunctive therapy for psychotropic medication–induced metabolic disturbances [56].

Given the favorable outcomes of GLP-1RAs—including weight loss, glycemic control, and additional cardiovascular and renal benefits in patients with obesity [57-59]—these agents should be prioritized for weight management in psychiatric populations. The dual challenges of obesity and psychiatric illnesses necessitate comprehensive treatment strategies. Initiating GLP-1RA therapy in high-risk individuals, particularly those taking antipsychotic or antidepressant medications associated with weight gain, may be an effective preventive approach that warrants further investigation, as suggested in previous guidelines [60].

Importantly, the weight loss observed in our systematic review was less pronounced than that reported in populations with obesity but without psychiatric comorbidities [14, 16, 18, 61, 62]. This discrepancy may be attributed to shorter trial durations and concurrent use of medications, such as clozapine or olanzapine, which are known to promote weight gain, and the influence of antidepressants. For example, a retrospective study found that GLP-1RA-induced weight loss was attenuated in patients concurrently using antidepressants, such as citalopram, escitalopram, and bupropion [63]. Similar findings have been observed in patients with type 2 diabetes, in whom underlying psychiatric illnesses may reduce the weight-lowering efficacy of GLP-1RAs [64].

This meta-analysis has some limitations. The heterogeneity of the included psychiatric conditions may limit the validity of indirect comparisons, as these disorders differ in terms of severity, treatment protocols, and levels of care required. Additionally, the limited number of studies available on this population constrains the strength of evidence. To date, only 1 RCT has evaluated oral semaglutide [32], and none have assessed tirzepatide, the most potent dual intestinal peptide agonist currently available for weight reduction. The results from 3 ongoing RCTs investigating subcutaneous semaglutide in psychiatric populations are awaited and may provide more definitive insights [65, 66] (NCT05333003).

## Conclusion

In summary, our findings demonstrate that GLP-1RAs significantly reduce body weight, BMI, waist circumference, and fasting glucose levels in patients with psychiatric disorders. Given their metabolic and potential neurobehavioral benefits, GLP-1RAs represent a promising therapeutic strategy, particularly in patients at high risk for metabolic complications. Future studies should explore their preventive use alongside psychotropic medications and investigate newer agents, such as tirzepatide, in this population, ideally through well-powered, long-term RCTs employing standardized protocols and rigorous adverse event reporting.

## Data Availability

The original contributions presented in this study are included in the article/supplementary material. Further inquiries can be directed to the corresponding author.

## Clinical Trial Information

Trial registration number: CRD42023418437 (registered on May 5, 2023).
